# A systematic review of psychosocial interventions for family carers of palliative care patients

**DOI:** 10.1186/1472-684X-9-17

**Published:** 2010-08-05

**Authors:** Peter L Hudson, Cheryl Remedios, Kristina Thomas

**Affiliations:** 1 Centre for Palliative Care c/o St Vincent's Hospital and The University of Melbourne, Australia and Queen's University, Belfast, UK

## Abstract

**Background:**

Being a family carer to a patient nearing the end of their life is a challenging and confronting experience. Studies show that caregiving can have negative consequences on the health of family carers including fatigue, sleep problems, depression, anxiety and burnout. One of the goals of palliative care is to provide psychosocial support to patients and families facing terminal illness. A systematic review of interventions for family carers of cancer and palliative care patients conducted at the start of this millennium demonstrated that there was a dearth of rigorous inquiry on this topic and consequently limited knowledge regarding the types of interventions likely to be effective in meeting the complex needs of family carers. We wanted to discern whether or not the evidence base to support family carers has improved. Furthermore, undertaking this review was acknowledged as one of the priorities for the International Palliative Care Family Carer Research Collaboration http://www.centreforpallcare.org.

**Methods:**

A systematic review was undertaken in order to identify developments in family carer support that have occurred over the last decade. The focus of the review was on interventions that targeted improvements in the psychosocial support of family carers of palliative care patients. Studies were graded to assess their quality.

**Results:**

A total of fourteen studies met the inclusion criteria. The focus of interventions included psycho-education, psychosocial support, carer coping, symptom management, sleep promotion and family meetings. Five studies were randomised controlled trials, three of which met the criteria for the highest quality evidence. There were two prospective studies, five pre-test/post-test projects and two qualitative studies.

**Conclusions:**

The systematic review identified a slight increase in the quality and quantity of psychosocial interventions conducted for family carers in the last decade. More rigorous intervention research is required in order to meet the supportive care needs of family carers of palliative care patients.

## Background

Being the family carer to a patient nearing the end of their life can be a challenging and confronting experience. The responsibilities of care may include complex physical and medical tasks, financial administration, patient advocacy, decision making, emotional support and coordination of care [1]. Furthermore, studies show that caregiving can have negative consequences on the health of family carers including fatigue, sleep problems, depression, anxiety, burnout and an increased risk of mortality [2,3]. Family carers of palliative care patients not only face the demands associated with caregiving, but also the grief and loss associated with their relative's impending death [4].

One of the goals of palliative care is to provide psychosocial support to patients and families facing terminal illness [5]. Psychosocial care has been defined as 'concern with the psychological and emotional well-being of the patient and their family/carers, including issues of self-esteem, insight into an adaption to illness and its consequences, communication, social functioning and relationships' [6].

Studies show that carers report the need for high levels of information and psychosocial support [7,8]. While unmet carer needs are widely recognised in the caregiving literature, there is limited knowledge regarding the types of interventions likely to be effective in meeting these complex needs [9]. One of the earliest systematic reviews of interventions for family carers of cancer and palliative care patients was conducted by Harding and Higginson [10]. Key databases were searched for reported interventions from 1966 to 2001. Their review identified 22 papers related to interventions. Five interventions were evaluated via randomised controlled trials. A range of intervention approaches was identified including home care, respite care, social networks and activities, problem-solving and education, one-to-one therapy and group work. Harding and Higginson [10]concluded that there was only a small body of evidence regarding the effectiveness of interventions for carers of cancer and palliative care patients; the bulk of the evidence came from a small number of studies that were graded as moderate to weak in terms of rigour.

Given that a paucity of interventions for family carers was identified at the start of this millennium, we wanted to discern whether or not the evidence base to support family carers has improved. Furthermore, evaluating the status of supportive interventions for carers was acknowledged as one of the priorities for the International Palliative Care Family Carer Research Collaboration http://www.centreforpallcare.org. Hence, we undertook a systematic review in order to identify developments in family carer support that have occurred over the last decade. The focus of the review was on interventions that targeted improvements in the psychosocial support of family carers of palliative care patients.

## Method

A systematic search of the literature was undertaken to identify family carer interventions for the period of January 2000 to July 2009. Electronic databases (Medline, CINAHL, EMBASE and PsychINFO) were searched. Cochrane data bases were examined separately. The following search term pathways were employed in electronic searches: Palliative care/terminal care/hospice AND carers/family/grief/death/bereavement AND support/interventions/therapy.

The following inclusion criteria were adopted in the current review:

(1) English language publications; (2) study populations involving adult family carers of palliative care patients. (For the purposes of this review we defined palliative care patients as people with life-threatening, advanced, incurable disease); (3) studies of psychosocial and/or psycho-education based interventions pre-patient death and (4) studies published between years 2000 to 2009.

Studies were excluded for the following reasons: (1) interventions that were patient-focused rather than carer-focused; (2) interventions designed to specifically support carers during their bereavement; (3) interventions based on complementary therapies such as massage and (4) interventions involving carers of patients with non life-threatening disease or potentially life-threatening disease. Publications that described the interventions were reviewed independently by two researchers and then crosschecked.

To remain consistent with Harding and Higginson's [10] review, studies were evaluated according to the same grading system [11]. The evidence was graded according to the rigour of study design and analysis (see Table 1). In keeping with Harding and Higginson's review interventions that were evaluated by qualitative methods were graded as weak evidence. Qualitative approaches to examining interventions were also included to assist in reporting on the evolution of this field of inquiry: we believe that clinicians and researchers will benefit from being aware of interventions even if they are only in the early stages of development. The importance of piloting interventions (to test processes, incorporating qualitative methods) for complex interventions is becoming increasingly evident [12,13]. We also included multicomponent interventions which incorporated educational approaches to improve family carers' management of patient symptoms because carers' psychological burden may be lessened if they are able to assist in improving their relatives' comfort [14].

**Table 1:** Grading criteria for review of carer intervention studies

**Grade I **(Strong evidence)
RCTs or review of RCTS
IA Calculation of sample size and accurate standard definition of appropriate outcome variables
IB Accurate and standard definition of appropriate outcome variables
IC Neither of the above

**Grade II **(Fairly strong evidence)
Prospective study with a comparison group (non-randomised controlled trial, good observational study or retrospective study that controls effectively for confounding variables).
IIA Calculation of sample size and accurate, standard definition of appropriate outcome variables and adjustment for the effects of important confounding variables
IIB One or more of the above

**Grade III **(Weaker evidence)
Retrospective or observational studies
IIIA Comparison group, calculation of sample size, accurate and standard definition of appropriate outcome variables
IIIB Two or more of the above
IIIC None of these

**Grade IV **(Weak evidence)
Cross-sectional study, Delphi exercise, consensus of experts

## Results

The total number of papers identified was 713. A review of the abstracts revealed that the overwhelming majority of these were related to the experience of caregiving and the needs of carers rather than the evaluation of specific interventions. Following the removal of 103 duplicates and a manual search for relevance, 25 papers were found to be related to interventions for family carers of palliative care patients. Of these, a total of 10 were omitted from our review as the focus of the interventions was on bereavement outcomes [15,16]; service utilisation [17,18] or patient outcomes [19-23].

A total of fourteen studies were therefore selected for the purposes of this review. These are presented in grading order (highest to lowest) in an additional file 1: "Intervention studies for family carers of palliative care patients published between 2000 - 2009". The goals of interventions included psychosocial support, psycho-education, carer coping, training in patient care (symptom management), sleep promotion and family meetings. Five studies were randomised controlled trials, two graded as good quality [24,25] and three that met criteria for the highest quality graded evidence in design [26-28]. There were two prospective studies with comparison groups that met criteria for fairly strong levels of graded evidence [29,30]. However, both these studies had relatively small family carer sample sizes. Five studies were pre-test/post-test designs without comparison groups and all were graded weaker in evidence [31-35]. Finally, two studies were examined via qualitative methods (vis semi-structured interviews) and were therefore graded as weak evidence [36,37].

All three of the randomised controlled trials with the highest graded evidence, evaluated interventions that focused on providing psychosocial support to enhance family carers' well-being [26-28]. A psycho-educational program for family carers was demonstrated to have a significant, favourable effect on carers' perceptions of the positive elements of their role [28]. A support intervention was found to improve family carers' quality of life, their perceived burden of patients' symptoms and their perceived burden of care tasks [26]. However, an evaluation of a separate psychosocial support intervention indicated no significant benefit to carers in the intervention group [27].

Two randomised controlled trials were graded slightly lower in evidence as they did not report use of power calculations [24], [25]. Nevertheless, both studies provide good quality evidence that interventions benefited family carers. One study showed that a partner-guided pain management training intervention was associated with significantly higher ratings of carer self-efficacy for helping patients control pain and other symptoms [24]. Another study of a counselling and support group intervention also showed positive effects on depression levels of carers of patients with Alzheimer's disease [25].

Of the two prospective intervention studies with comparison groups, only one showed significant benefit to family carers. Carter [29] found that a brief behavioural sleep intervention produced greater improvements in sleep quality and depression in the carer intervention group compared to the control group. However, Harding et al. [30] reported that a short-term intervention promoting self-care had no significant benefit to carers' psychosocial health or well-being. However a qualitative study with family carers who participated in the intervention revealed that valued outcomes were validation of feelings, identification with other carers, opportunities for questions and provision of support to others [36].

The pre-test/post-test studies without comparison groups all showed favourable results of the interventions. Hudson et al. [32] reported that a psycho-educational group program had significant positive effects on carers' preparedness, competence, reward ratings and reduced unmet needs. Hudson et al. [33] also found that a family meeting intervention reduced unmet family carer needs. Kwak et al. [34] reported a significant increase in carers' levels of comfort, closure and satisfaction following the attendance of a carer support program. Lastly, Walsh and Schmidt [35] found that a telephone support intervention had psychological benefits for participating carers. However, due to attrition the data were based on a sample of only five carers. The aforementioned studies were graded weaker in evidence due to the lack of comparison groups.

Two studies evaluated via qualitative data methods reported that carers perceived psychosocial support interventions as beneficial. Duggleby et al. [31] conducted a pre-test/post-test study of a program intended to promote hope among carers. While the study sample size precluded statistical analyses the qualitative responses indicate the program was received favourably. Milberg et al [37] found that regular support group sessions for family carers of palliative care patients were also perceived as beneficial when follow-up evaluations were conducted. All participants reported that they would recommend a support group to others in a similar situation.

## Discussion

The evaluation of interventions for family carers of palliative care patients conducted in the last decade shows a slight increase in the quality and quantity of psychosocial care strategies for carers. In Harding and Higginson's review only one intervention met the highest level of grading [10]; whereas we identified three studies that met this criteria. Nonetheless, as Harding and Higginson acknowledged, meta- analyses still do not seem feasible given the heterogeneity in study outcomes and design.

Three randomised controlled trials showed that psycho-educational support interventions had beneficial outcomes for family carers. This reinforces Eager and colleagues claim (based on a review of interventions across a variety of carer groups) that interventions aimed towards psycho-education, problem-solving and cognitive restructuring can show demonstrable effects on carer well-being [13].

Several studies with favourable treatment effects utilised interventions that targeted specific needs of carers such as therapy for sleep deprivation and training in problem-solving skills. This finding is commensurate with other authors who have advocated the importance of targeted interventions for this population [38].

Studies with lower graded evidence also showed favourable trends in outcomes of interventions for carers. While these designs were limited by less rigorous approaches, such studies will potentially provide the basis for the future development of higher quality designs. Several of these studies examined novel intervention approaches for end-of-life carers such as facilitation of family meetings and pain management. The development of these and other new intervention approaches is vitally important to the evolution of this field of inquiry. We concur with others who have recognised the crucial role that pilot and descriptive intervention projects have prior to launching into large randomised controlled trials [12].

Our review has also revealed that the majority of interventions seem to be conducted in first world countries. The applicability of these approaches to other cultures and contexts therefore requires further investigation.

Recommendations from two recent reviews of research related to family caregivers may assist with future intervention design. Grande and colleagues [9] recommend that future work in this area should focus on the following: clear definition and operation of intervention goals; separate and specific assessment of carers needs; greater focus on preventative intervention approaches; facilitation of the positive aspects of caregiving; development of valid and reliable measures in carer assessment; and better understanding of the 'active' components of interventions.

Eager and colleagues [13] concluded from their review of interventions for family carers across a variety of patient groups that they had difficulty yielding conclusive information on the effectiveness of carer support interventions. They argued that there was little known about the effective 'dose' of support interventions or the best time for their delivery. Possible explanations offered for interventions found to be ineffective were inappropriate outcome measures; non-specific goals that do not target needs; and ineffective research evaluation designs [13,39]. Also, interventions based on an individual approach were regarded as more likely to have significant effects for carer burden and well-being whereas group approaches typically worked well for building carer competence. Eager and colleagues [13] also contend there is minimal evidence to suggest that the needs of family carers systematically vary based on the type of person being cared for. This recommendation supports the view put forward by others [40] who argue that there may be ways of designing interventions for supporting carers that have generic application, whilst acknowledging there will always be the need for individual variation.

The aforementioned recommendations are useful in assisting with the future development of psychosocial interventions for family cares. However several key questions remain: First, how can psychosocial interventions be designed to be effective given the typically short period of time available to intervene? Second, what is the most useful way to determine which family carers need significant psychosocial support? Third, how can health services meet the support needs of the entire family when many may only be resourced to support the primary family carer? And finally what are the priority interventions and methods of delivery that are required for development and testing in the family carer population?

## Conclusion

This systematic review of psychosocial interventions for family carers of palliative care patients has identified that the empirical basis for discerning the types of strategies that help carers has improved slightly over the last decade. Hence, there are still significant improvements to be made in terms of the number, rigour and design of future studies. Ten years on, we concur with Harding and Higginson's conclusion that empirical inquiry regrading effective ways to provide psychosocial support to family carers is still in its infancy. Unless this matter is redressed there will continue to be a disconnect between what is advocated in policy (that family carers needs are assessed and adequately responded to) and what actually happens in clinical practice (health professionals operating without a suitable evidence base to support family carers).

This review has several limitations: interventions that targeted family caregivers during bereavement and those published in languages other than English were not included. Nonetheless, based on this review we contend that unless there is a major research investment in this area the claim that palliative care services provide family carers with effective support will continue to be disputed [41].

## Competing interests

The authors declare that they have no competing interests.

## Authors' contributions

PH was the principal investigator for the study. He oversaw management of the project and was responsible for drafting the manuscript and preparing revisions and the final submission. CR was a chief investigator and assisted with design, data collection and provided input into the manuscript focusing on the literature review. KT was a project officer and helped conceptualise the study design and provided feedback on draft versions of the manuscript. All authors read and approved the final manuscript.

## Pre-publication history

The pre-publication history for this paper can be accessed here:

http://www.biomedcentral.com/1472-684X/9/17/prepub

## Supplementary Material

Additional file 1 **Intervention studies for family carers of palliative care patients published between 2000-2009**. File contains a table that runs over three pages; it is to be inserted in text prior to Discussion. Click here for file

## References

[B1] Stajduhar K Cohen R Hudson P, Payne S Family caregiving in the home Family Carers and Palliative Care 2009 Oxford University Press

[B2] Schulz R Mendelsohn AB Haley WE Mahoney D Allen RS Zhang S Thompson L Belle SH End of life care and the effects of bereavement on family caregivers of persons with dementia New England Journal of Medicine 2004 349 20 1936 1942 10.1056/NEJMsa035373 14614169

[B3] Thomas C Morris M Harman J Companions through cancer: the care given by informal carers in cancer contexts Social Science Medicine 2002 54 529 544 10.1016/S0277-9536(01)00048-X 11848273

[B4] Candy B Jones L Williams R Tookman A King M Interventions for supporting informal caregivers of patients in the terminal phase of a disease (Protocol) Cochrane Database of Systematic Review 2009 1 10.1002/14651858.CD007617.pub2 PMC13247825 21678368

[B5] Macleod Lloyd-Williams M Setting the context: what do we mean by psychosocial care in palliative care? Psychosocial issues in palliative care 2008 New York: Oxford University Press

[B6] NCHSPCS Feeling better: psychosocial care in specialist palliative care 1997 13 London: National Council for Hospice and Specialist Palliative Care Services

[B7] Andershed B Relatives in end-of-life care -- part 1: a systematic review of the literature the five last years, January 1999-February 2004 Journal of Clinical Nursing 2006 15 9 1158 1169 (1111 ref) 10.1111/j.1365-2702.2006.01473.x 16911057

[B8] Aoun SM Kristjanson LJ Hudson PL Currow DC Rosenberg JP The experience of supporting a dying relative: reflections of caregivers Progress in Palliative Care 2005 13 6 319 325 10.1179/096992605X75930

[B9] Grande G Stajduhar K Aoun S Toye C Funk L Addington-Hall J Payne S Todd C Supporting lay carers in end of life care: current gaps and future priorities Palliative Medicine 2009 23 339 344 10.1177/0269216309104875 19304804

[B10] Harding R Higginson I What is the best way to help caregivers in cancer and palliative care? A systematic literature review of interventions and their effectiveness Palliative Medicine 2003 17 1 63 74 10.1191/0269216303pm667oa 12597468

[B11] Cancer Guidance Subgroup of the Clinical Guidance Outcome Group. Improving outcomes in breast cancer- the research evidence Leeds: NHS Executive 1996

[B12] Craig P Dieppe P Macintyre S Michie S Nazareth I Petticrew M Developing and evaluating complex interventions: the new Medical Research Council guidance British Medical Journal 2008 337 a1655 18824488 10.1136/bmj.a1655 PMC2769032

[B13] Eagar K Owen A Williams K Westera A Marosszeky N England R Morris D Effective Caring: a synthesis of the international evidence on carer needs and interventions 2007 Centre for Health Service Development, University of Wollongong

[B14] Kristjanson L Aoun S Palliative Care for Families: Remembering the hidden patients Canadian Journal of Psychiatry 2004 49 6 359 365 10.1177/070674370404900604 15283530

[B15] Holland J Currier JM Gallagher-Thompson D Outcomes from the resources for enhancing alzheimer's caregiver health (REACH) program for bereaved caregivers Psychol Aging 2009 2009 24 1 10.1037/a0014303 19290751

[B16] Kissane DW McKenzie M Block S Moskowitz C McKenzie DP O'Neill I Family Focused Grief Therapy: A Randomized, Controlled Trial in Palliative Care and Bereavement American Journal of Psychiatry 2006 163 7 1208 1218 10.1176/appi.ajp.163.7.1208 16816226

[B17] Dobrof J Ebenstein H Dodd S Epstein I Caregivers and Professionals Partnership Caregiver Resource Center: Assessing a Hospital Support Program for Family Caregivers Journal of Palliative Medicine 2006 9 1 196 205 10.1089/jpm.2006.9.196 16430359

[B18] Phillips JL Davidson PM Newton PJ DiGiacomo M Supporting Patients and Their Caregivers After-Hours at the End of Life: The Role of Telephone Support Journal of Pain and Symptom Management 2008 36 1 11 21 10.1016/j.jpainsymman.2007.08.017 18411012

[B19] Lily CM De Meo DL Sonna LA Haley KJ Massaro AF Wallace RF Cody S An intensive communication intervention for the critically ill The American Journal of Medicine 2000 109 469 475 10.1016/S0002-9343(00)00524-6 11042236

[B20] Clayton JM Butow P Tattersall MHN Devine R Simpson J Aggarwal G Clark K Currow D Elliott L Lacey J Randomized controlled trial of a prompt list to help advanced cancer patients and their caregivers to ask questions about prognosis and end of life care Journal of Clinical Oncology 2007 25 6 715 723 10.1200/JCO.2006.06.7827 17308275

[B21] Schneiderman L Gilmer T Teetzel H Dugan D Blustein J Cranford R Briggs K Komatsu G Goodman-Crews P Cohn F Effect of ethics consultations on nonbeneficial life-sustaining treatments in the intensive care setting: a randomized control study JAMA: Journal of the American Medical Association 2003 290 9 1166 1172 10.1001/jama.290.9.1166 12952998

[B22] Grande G Todd C Barclay S McFarquhar A randomized controlled trial of a hospice at home service for the terminally ill Palliative Medicine 2000 14 375 385 10.1191/026921600701536200 11064784

[B23] Clark MM Rummans TA Sloan JA Jensen A Atherton PJ Frost MH Richardson JW Bostwick JM Johnson ME Hanson JM Quality of life of caregivers of patients with advanced-stage cancer American Journal of Hospice & Palliative Medicine 2006 23 3 185 191 10.1177/1049909106289074 17060277

[B24] Keefe FJ Ahles TA Sutton L Dalton J Bauchom D Pope M Knowles V Mc Kinstry E Furstenberg C Syrjala K Partner-guided cancer pain management at the end of life: a preliminary study Journal of Pain and Symptom Management 2005 29 3 263 272 10.1016/j.jpainsymman.2004.06.014 15781177

[B25] Haley WE Long-term effects of bereavement and caregiver intervention on dementia caregiver depressive symptoms The Gerontologist 2008 48 6 732 740 19139247 10.1093/geront/48.6.732 PMC2846300

[B26] McMillan SC Small BJ Weitzner M Schonwetter R Tittle M Moody L Haley WE Impact of coping skills intervention with family caregivers of hospice patients with cancer: A randomized clincial trial Cancer 2006 106 1 214 222 10.1002/cncr.21567 16329131

[B27] Walsh K Jones L Tookman A Mason C McLoughlin J Blizard R King M Reducing emotional distress in people caring for patients receiving specialist palliative care British Journal of Psychiatry 2007 190 142 147 10.1192/bjp.bp.106.023960 17267931

[B28] Hudson PL Aranda S Hayman-White K A psycho-educational intervention for family caregivers of patients receiving palliative care: a randomised controlled trial Journal of Pain & Symptom Management 2005 30 4 329 341 10.1016/j.jpainsymman.2005.04.006 16256897

[B29] Carter P A brief behavioral sleep intervention for family caregivers of persons with cancer Cancer Nursing 2006 29 2 95 103 10.1097/00002820-200603000-00003 16565618

[B30] Harding R Higginson I Leam C Donaldson N Pearce A George R Robinson V Taylor L Evaluation of a short term group of intervention for informal carers of patients attending a home palliative care service Journal of Pain and Symptom Management 2004 27 5 396 408 10.1016/j.jpainsymman.2003.09.012 15120768

[B31] Duggleby W Wright K Williams A Degner L Cammer A Holtslander L Developing a living with hope program for caregivers of family members with advanced cancer Journal of Palliative Care 2007 23 1 24 31 17444459

[B32] Hudson P Thomas K Quinn K Cockayne M Braithwaite M Teaching family carers about home based palliative care: final results from a group education program Journal of Pain and Symptom Management 2009 38 2 299 308 10.1016/j.jpainsymman.2008.08.010 19345553

[B33] Hudson P Quinn K O'Hanlon B Aranda S Family meetings in palliative care: multidisciplinary clinical practice guidelines BMC Palliative Care 2008 7 12 18710576 10.1186/1472-684X-7-12 PMC2542352

[B34] Kwak J Salmon JR Acquaviva KD Brandt K Egan KA Benefits of training family caregivers on experiences of closure during end-of-life care Journal of Pain & Symptom Management 2007 33 4 434 445 10.1016/j.jpainsymman.2006.11.006 17397704

[B35] Walsh S Schmidt L Telephone support for caregivers of patients with cancer Cancer Nurs 2003 26 6 448 453 10.1097/00002820-200312000-00004 15022976

[B36] Harding R Leam C Pearce A Taylor E Higginson IJ A multi-professional short-term group intervention for informal caregivers of patients using a home palliative care service Journal of Palliative Care 2002 18 4 275 281 12611318

[B37] Milberg A Rydstrand K Helander L Friedrichsen M Participants' experiences of a support group intervention for family members during ongoing palliative home care Journal of Palliative Care 2005 21 4 277 284 16483097

[B38] McCorkle R Pasacreta V Enhancing Caregiver Outcomes in Palliative Care Cancer Control 2001 8 1 36 45 11252271 10.1177/107327480100800106

[B39] Ducharme F Lebel P Lechance L Trudeau D Implementation and effects of an individual stress managment intervention for family caregivers of an elderly relative living at home: a mixed search design Research in Nursing & Health 2006 29 5: 10.1002/nur.20152 16977641

[B40] Hudson P Payne S Hudson P, Payne S The future of family caregiving: research, social policy and clinical practice Family Carers in Palliative Care: A guide for health and social care professionals 2009 Oxford: Oxford University Press 277 303

[B41] Harding R Firth P, Luff G, Oliviere D Carers: Current research and developments Facing Death: Loss, Change and Bereavement in Palliative Care 2005 1 Maidenhead, Berkshire: Open University Press 150 166

