# Supplementary material for: A systematic review of psychosocial interventions for family carers of palliative care patients
Source: BMC Palliat Care. 2010 Aug 5;9:17. doi: 10.1186/1472-684X-9-17 (PMC2924287; doi:10.1186/1472-684X-9-17)
Supplement: Additional file 1 — Intervention studies for family carers of palliative care patients published between 2000-2009. File contains a table that runs over three pages; it is to be inserted in text prior to Discussion. [file 1472-684X-9-17-S1.DOC]

**Intervention studies for family carers of palliative care patients published between 2000 - 2009**

| **Reference and**  **grade of evidence** | **Study population** | **Intervention description** | **Evaluation design** | **Carer Outcome variables** | **Findings** |
| --- | --- | --- | --- | --- | --- |
| McMillan et al. [26]  IA | 354 family carers of community palliative care patients | COPE Intervention: problem-solving/coping skills intervention with four components: creativity, optimism, planning and expert information | RCT following inclusion assessment. Trial arms were (1) Usual care group (n=109); (2) Usual care + three support visits (n=109); and (3) Usual care + 3 visits to teach intervention (n=111). Data collection was at baseline, one wk post-intervention and two wks post-intervention | *Caregiver quality of life  * Caregiver mastery  *Caregiver demands | At 30-day follow-up, the intervention showed significantly improved quality of life, burden of patients’ symptoms and caregiving task burden compared to the other two conditions. There were no changes in caregiving mastery or coping. |
| Hudson et al [28]  IA | 106 carers of patients receiving home-based palliative care | A psycho-educational intervention delivered via two home visits and two phone calls by a nurse. This was supplemented with a guide book and audio tape. Program content included preparation, information, carer needs, psychosocial support, respite, future planning and goals. | RCT following eligibility assessment. Trial arms were (1) standard palliative care and (2) standard care + intervention. Data collected at baseline, five weeks later and eight weeks following patient death. | *Preparedness for caregiving  *Caregiver competence  *Rewards of caregiving  *Anxiety and depression  *Mastery  *Self-efficacy | A significant positive effect of the intervention was found in perceptions of caregiving rewards. No other significant treatment effects were found. |
|  |  |  |  |  |  |
| Walsh et al. [27]  IA | 271 informal carers of patients in palliative care | Six visits by carer advisors to meet needs of carers and provide additional support to palliative care services. Domains covered: patient care, physical health needs, respite, future planning, psychosocial issues, relationships with health professionals and finances | RCT following baseline assessment. Trial arms were usual care from specialist palliative care service (n=134) and intervention (n=137). Follow-up was at 4, 9 and 12 weeks. | *Psychological distress  *Carer strain  *Carer quality of Life  *Bereavement assessment | Scores on GHQ fell below the threshold of 5/6 in a third of carers in both arms at all follow-up points. No significant differences between groups were found on any outcome measures. |

| **Reference and**  **grade of evidence** | **Study population** | **Intervention description** | **Evaluation design** | **Carer Outcome variables** | **Findings** |
| --- | --- | --- | --- | --- | --- |
| Keefe et al [24]  IB | 78 partners of advanced cancer patients eligible for hospice care | A cognitive-behavioural, partner-guided pain management training intervention over three (45 to 60 min) sessions in patients’ homes. The aims of the intervention were (1) education, (2) pain management and (3) coping skill maintenance. Written materials, a videotape and audiotapes were also provided. | RCT Following a pre-treatment phone assessment, patients and partners were randomly assigned to either the intervention or standard care. Post-treatment evaluations were conducted by phone and/or mail a mean of 7.56 days following completion. | *Functional assessment  *Self-efficacy in chronic pain assessment  *Mood | Partners who received the intervention reported significantly higher levels of self-efficacy for helping the patient control pain and other symptoms. There was non-significant trend for partners to report lower levels of carer strain. |
| Haley et al. [25]  IB | 254 spousal carers of Alzheimer’s patients who had experienced the death of their spouse during participation in the New York University Carer Intervention Project | Carer intervention included (a) 2 individual and 4 family counselling sessions; (b) a weekly support group; and (c) Ad-hoc counselling upon request. The usual care arm received standard care, which included counselling upon request. | RCT Prior to death of spouse, carers were randomly assigned to the intervention (n=122) or usual care (n=132) and baseline measures were taken. Participants were followed up every 4-6 months prior to patient death and either one or two years after patient death. | * Depression | Carers in intervention group had significantly lower depression symptoms compared to those in usual care both before and after bereavement. These effects were more marked among carers whose spouses were not nursing home placed. |
| Carter [29]  IIB | 30 adult carers of advanced cancer patients with at least a six month life expectancy who were not enrolled in hospice services | A brief behavioural sleep intervention for family carers that included stimulus control, relaxation, cognitive therapy and sleep hygiene training in two one hour sessions. The control group received body mechanics training in two one hour sessions. | Repeated measures experimental design with intervention group (n=15) and control group (n=15). Data collected at baseline, three and five weeks, and two, three, and four months post baseline. | * Sleep quality  *Sleep monitoring  *Sleep logs  *Depression  *Carer quality of life | Improvement in both groups but intervention group showed greater improvements in sleep quality and depression scores than controls. Quality of life scores were similar across groups. |

| **Reference and**  **grade of evidence** | **Study population** | **Intervention description** | **Evaluation design** | **Carer Outcome variables** | **Findings** |
| --- | --- | --- | --- | --- | --- |
| Harding et al. [30]  IIB | 73 family carers of patients receiving home-based palliative care. | Short-term intervention in group format aimed to promote self-care by combining informal teaching with group support in six weekly sessions of 90 minute duration. | Pre-post design: Intervention (n=36) and comparison group (n = 37)  Three time points: baseline, 8 wks from baseline and 5 months from baseline  Semi-structured interviews at 8 weeks from baseline | *Palliative outcomes  *Patient performance  *Burden  *Coping  *General health  *State anxiety | Group intervention had no significant effect on any outcome variables post-intervention or at follow-up. Qualitative data showed carers valued talking to others and sharing experiences |
| Hudson et al. [33]  IIIB | 20 family carers of patients in palliative care, 18 health professionals and 4 patients | Nurses received training on the facilitation of family meetings based on clinical practice guidelines. Family meetings were conducted with carers and follow-up phone calls were made. | Pilot study: Pre-post survey design. Three time points: before meeting, after meeting and two days after meeting. | *Family needs  *Pre/post meeting questionnaire  *Family meeting evaluation forms  *Qualitative data from focus groups | Carers reported a significant increase in having their needs met from TI to T2 and T3. Professionals and patients reported meetings were well facilitated. |
| Hudson et al. [32]  IIIB | 156 primary family carers of patients with advanced cancer receiving palliative care | Carer Group Education Program: a psycho-educational intervention (three sessions over three weeks) that covered (1) the carer role; (2) strategies for self-care; and (3) strategies for patient care; delivered by health professionals. | Pre-post survey design  Three time points: commencement of program, upon completion and two weeks later. | *Carer competence  *Preparedness for caregiving  *Family needs  *Rewards of caregiving | Significant positive effects of the intervention were found for caregiving preparedness, competence, rewards and unmet needs. |
| Kwak et al. [34]  IIIB | 2025 end-of-life family carers | The Hospice Experience Model of Care program was delivered by 142 trainers in five sessions. The program included modules on: life affairs; community relationships; personal relationships; love; acceptance of end-of-life; and bereavement. | Pre-post survey design: Pre-surveys were administered at the end of the first session and post-surveys were completed at the end of the last session. | *Comfort with caregiving  *Carer closure  *Carer satisfaction | Significant improvement was found in all three outcomes. Program length made a difference for improvement in comfort with caregiving and closure but not in carer gain. |

| **Reference and**  **grade of evidence** | **Study population** | **Intervention description** | **Evaluation design** | **Carer Outcome variables** | **Findings** |
| --- | --- | --- | --- | --- | --- |
| Duggleby et al.  [31]  IIIC | 10 family carers of people with advanced cancer | Living with Hope program delivered via a home visit. The program consists of a 17- minute video and a daily journaling activity. | A mixed method concurrent triangulation, pre-and post-test design. Quantitative and qualitative data collected at baseline, prior to program delivery. Data also collected at two post-treatment time points (one and two weeks post-treatment). | * Hope  *Quality of life  *Qualitative open-ended evaluation questions | The sample size precluded statistical analyses although mean scores on both measures did increase. Qualitative data was said to be very positive in evaluation of the program. |
| Walsh & Schmidt  [35]  IIIC | 14 carers of community palliative care patients recruited but only five completed study due to patient death | A four week telephone support intervention modified for carers of hospice patient. Based on ‘tele-care II intervention, which uses a tele-care workbook to provide information related to palliative care. The information was delivered via teleconferencing with a nurse. | Pre-post study. Data collected prior to intervention and post intervention (if patient had not died). | *Caregiver burden  *depression  *social support  *end-of-life reactions | Carers who completed the full study were found to experience decreased depression, despair and disorganisation as the patient’s condition worsened. |
| Harding et al.  [36]  IV | 40 family carers of home based palliative care patients participated but only 21 completed evaluations | As described in Harding et al. [30] (above) | Qualitative study with semi-structured interviews | Topics in interviews addressed motivation for attending, format, participation, content, information and benefits gained. | Attendees reported that identifying with other carers, validating feelings, asking questions, and providing support were valuable outcomes. |
| Milberg et al.  [37]  IV | 19 family carers of patients receiving palliative home care | Weekly support groups run over six or seven weeks for approximately 1.5 hours. All led by health professionals (social worker and physiotherapist) with prepared topics such as ‘psychological responses’ and ‘community resources’; but the aim was to promote flexibility in discussion topics. | Follow-up evaluations of the support group were conducted via four tape-recorded focus group interviews (13 members participated) and a questionnaire (completed by 19 members). | Interview questions regarding perceptions of support group. Thematic analysis of focus group data. | The majority of carers reported favourably on participation in focus groups and recommend it to other carers. Themes that emerged from focus groups were: reasons for group participation; group composition; group leader; meaningful dialogue; sense of cohesion; and post-session reflections. |
